# Mysterious faces of hybridisation: an anticipatory approach for crisis literacy

**DOI:** 10.1186/s40309-022-00207-5

**Published:** 2022-09-10

**Authors:** Joni Karjalainen, Sirkka Heinonen, Amos Taylor

**Affiliations:** Finland Futures Research Centre, University of Turku, Turku, Finland

**Keywords:** Anticipatory governance, Change, Crisis, Hybridity, Urban, VUCA world

## Abstract

Our complex world is changing at such a pace that we are struggling to address many of the global challenges ahead of us. As one of its symptoms, hybridisation means that fields, functions, characteristics and roles are increasingly combined and fused. This paper is an opening to the study of hybridisation, as an overlooked topic in the field of futures studies and foresight. We explore how hybridisation could be integrated into foresight through identification and interpretation of emerging issues and weak signals. As our case study, we examined how hybridisation manifests in the urban texture. We performed an anticipatory analysis of three hybrid urban spaces of pioneering architecture. We assumed a view to hybridity that considered diverse futures, images of the future, and open futures to detect what is opening or closing. Coming to terms with hybridisation and its expressions may inform action on anticipatory governance by improving the detection of opportunities, risks and crises. Deeper understanding of budding developments that removes ambiguity may be a nudge towards novel solutions and promote futures resilience.

## Introduction

This paper explores the concept of hybridisation, and how it embodies fusions of emerging issues, in order to open up a discussion on its role for futures studies and foresight. As a starting point, we claim that the field of futures studies has not looked deep enough into *hybridisation*, as an understudied area. Accelerating pace of change is fertile soil for risks and crises [18], which calls for broader knowledge and application of new concepts and frameworks to be adopted, so that unpredictable and rapid changing situations can be dealt with [36]. Recent crises seem to embody the characteristics of a ‘VUCA’ world (volatility, uncertainty, complexity, and ambiguity). The VUCA approach, which originates from the end of the Cold War era, United States Army War College, has been taken up in businesses and strategy planning [3, 29]^1^. From these premises, we claim that hybridisation is a phenomenon that is increasingly present in our societies.

A core motivation for this paper in analysing hybridisation stems from futures studies, and is concerned with the purpose of foresight, namely to scan for change at various levels and in all dimensions of society [40]. If modernity was about clearly demarcated and delineated domains, as argued by Latour [33, 34], hybridisation is about the mixtures of human and non-human, of nature and culture in quite complex ways. Already, it is difficult to grasp our world of ubiquitous digital technology [10], so what happens when a multitude of potential changes lie ahead of us. Drawing on ([54], 103-105), for Latour, hybridisation is about ‘amodernity’ (as he refutes the idea of modernity), whereas for Beck, it is about a ‘second form’ of modernity, as a move from simple to reflexive modernity, and into a risk society. Such issues point to a growth in complexity in societies.

Concerning the futures studies community, our motivating idea is to begin to explore how hybridisation is revealing itself as a growing number of combinations and fusions of things, aspects, elements and actors, to interpret and learn from them, including in relation to various global challenges and crises. With respect to foresight, our paper is a call to develop approaches, techniques and methods that can help make sense of hybridisation, with a view to emerging issues, weak signals, and their typically ambiguous nature. As our case study, we considered the expressions of hybridisation in the urban space. Urban spaces are a locus of multiple imaginations where futures are continuously explored, expressed and manifested [8]. In fact, urban scholars explain that the complexity and multi-faceted nature of cities owes to the multiple manifestations of hybridity, giving them their very nature [13]. Urban futures are imagined and co-evolve in the nexus of architecture, planning and visioning [9]. In endeavours addressed at the urban space, we manifest and exercise our anticipatory habits, or “socio-material organisations of anticipation” ([20], 30). In this paper, hybrid urban spaces are considered as engagement points where the socio-technical, the socio-material and the nature interact. We also project the role of citizens onto these venues pointing to futures [64].

The paper advances as follows. Hybridisation and its manifestations in different fields are opened up to de-mystify hybridisation in the second section. We dig deeper into an overlooked subject: what does it mean and where does this concept come from? From these premises, we argue that hybridisation becomes a useful pointer to anticipatory governance and crisis literacy. The third section presents our methodology and data on hybrid urban spaces as examples of pioneering architecture in the urban environment. In the fourth section, we probe how such hybrid spaces could protect citizens and cities from crises, provide resilience, and scan for vulnerabilities. In the fifth section, drawing on our analysis, we propose ways to integrate hybridity in futures studies and foresight, with a specific view to our case study of urban planning. We discuss how learning from hybridisation could enhance our preparation to futures, serve as a lever for the construction of futures, in identifying or alleviating crises, and in opening and closing futures. The sixth section concludes and raises questions for further studies.

## Hybridisation and anticipatory governance

‘Hybrid’ is interpreted somewhat differently in different fields. ‘Hybridity’, ‘hybridisation’, ‘hybridism’ or ‘hybrid’ are terms used by scholars in the social sciences as well as literary, artistic, and cultural studies. These designate processes in which discrete social practices and structures, that existed in separate ways, combine to generate new structures, objects, and practices in which such elements mix.

### Hybridisation—origin, definition, and manifestations

Hybridity is no novelty. It dates back to mythology, as ancient "power beast characters", and to biology, with “mixed creatures”. In Ancient Greece, mythical beasts such as Hydra were frightening figures of power, rather than intriguing creatures. In biology and chemistry, hybridisation equates to a ‘process of combining different varieties of organisms to create a hybrid’. We emphasise hybridisation as a multidisciplinary subject [59], with multiple interpretations, which is now re-emerging on many fronts with vigour and visibility. From the particular view of futures studies, the topic of hybridisation draws attention to change. Can change be anticipated better through analysing hybridisation? Does hybridisation accelerate or hinder change?

We adopt the term ‘hybridisation’ to emphasise the dynamic nature of multiple phenomena. To illustrate what we mean, accordingly:

- *Hybrids* are combinations;
- *Hybridity* is the phenomenon of the existence of such combinations; and
- *Hybridisation* emphasises the dynamic nature of increasing hybrids in society.

A hybrid can be based on many combinations, constituted of elements of very different scale or nature. As combinations of two or more things, hybrids can be categorised according to their (1) characteristics, (2) fields, (3) functions, and (4) roles (Table 1). Sometimes hybrids combine even seemingly incompatible characteristics. The presented combinatory elements, as examples, are naturally not exhaustive. More hybrids, even overlapping ones can be placed in each category.

**Table 1:** Four categories of hybrids as a typology

Hybrids as combinations of different **characteristics:**	Hybrids as combinations of different **fields:**
Physical + virtual/digital	Technology + culture/art
Public + private	Technology + nature
Tangible + intangible	Science + religion
Natural + artificial	Mathematics + music
Rational + emotional/spiritual	Health + agriculture
Mundane + celestial	Humans + machines
…	…
Hybrids as combinations of different **functions:**	Hybrids as combinations of different **roles:**
Work + housing	Citizen + leader
Work + mobility	Consumer + producer
Housing + mobility	Student + teacher
Learning + entertainment	Employer + employee
Communicating + caring	Volunteer + activist
Business + leisure	Authority/expert + layperson
…	…

Hybrid concepts have become colloquial through hybrid TVs (analogue + digital), hybrid cars (electronic + fuel), and hybrid work (home + office). The COVID-19 pandemic propelled hybrid strategies (testing, tracking, isolating, curing, and keeping society functioning). Hybrid warfare blends conventional warfare, irregular, asymmetric and cyberwarfare with influences, such as fake news, and foreign interference.

In cultural studies, the hybridisation of culture, identity and ethnicity explores the construction of individual and collective modern identities as a post-colonial or changed perspective or the double construction of the self, which implies a kind of ‘Hybrid Consciousness’ [43]. To Bruno Latour [33] modernity represents a dual process of “purification” and “hybridisation”, the latter referring to mixtures of nature and culture. Media studies recognise digital and virtual reality, human and machine hybridity, even cyborgs. The *‘Walkman effect’* explains how headphones and listening to music in the urban environment enabled anyone to immerse to the ‘soundtrack for their lives’, transforming the sense of reality [26]. A dual digital of online social media and smartphone applications embody how our sense of the ‘real’ world is changing, as it is overlaid, augmented, and merged with digital realities. The GPS systems for navigation and augmented games like ‘Pokémon Go’ have made players rush to physical locations where they collect virtual points. Wireless connectivity subverts the geographical space, offering freedom and mobility. More and more phenomena are hybridising our everyday life and spaces. These include the convergence of technologies, practices, and expressions of social mood [6], as unfolding complex phenomena.

Recent social-scientific views may further help us understand hybridity. In organisational studies, Minkoff [41] attends to *hybrid organisations*, while Billis [4] explores their emergence and diverse structural features to make sense of the ingredients that constitute them. Martens et al. [37] interpret hybridity as one feature of globalisation where human dynamics, institutional change, political relations and the global environment become increasingly intertwined. A near comparison to hybrids are assemblages, as collections of things that have been gathered together or assembled [46]. *Assemblage theory* is a distinct intellectual lineage, which argues that “reality is made up of relations between different elements, subjects, organizations, objects, immaterial and material, natural, cultural etc. that are organised in assemblages” ([58], 205). Assemblages are more about a philosophical view of relations and connectedness to a whole—an entity, whereas hybridisation, is about the question of fusion, merging together of different entities to form a whole, making the latter an interesting object of empirical analyses.

Hybridity, as the multi-layered aspects of reality and consciousness, is at play in the built environment also in times of crises. The clashes between protesters and authorities can transform everyday street corners and parks into urban battlegrounds. New technologies help organise secret assemblies, whereas everyday objects like umbrellas offer protection from mass surveillance. At the ‘Occupy’ movement, the financial epicentres of London and New York became live-in protest scenes, where tents and banners subverted the meaning of the space. The ‘Fridays for Future’ youth movement has forgone attending school by assembling at key public spaces every Friday. Similar scenes speak to a changing world by transforming the function, meaning and aesthetics of space.

From a futures studies perspective, the study of hybridisation is specifically interesting from the perspective of emergence. According to our view, these fusions express ambiguity of the VUCA world. Ambiguous mixtures pointing to a previous lack of distinct categories [37] make hybridity open and challenging to interpret, and give hybridity its mysterious character. The creation of multiple combinations shows itself in innovations and, as mentioned, in assemblages where potentially multiple, disparate elements are assembled [58]. However, it may be that especially structural kinds of fusions are those that may really begin to blur our understanding of the world. Generally, we are interested in any attempts to examine the phenomenon from the aspects in which it may help us better anticipate and understand possible futures, as part of efforts to improve our futures literacy [39].

### Anticipatory governance and crisis literacy

There are loud calls to overcome short-termist decision-making and policy-making, which usually have considered a set of slowly moving issues in the policy radar. Instead, governance should effectively “use the future” [38] by taking an agile, strategic, and long-termist stance toward futures by anticipating and engaging with emerging issues that often have a high degree of uncertainty. So far, the main advances to combine foresight and governance have been in outlining an agenda for anticipatory governance. Anticipatory governance (AG) is advocated as a capacity of elected officials to look ahead, envision, imagine, assess, strategize, deliberate, respond, and transform [15]. As a starting point, AG aims to apply and systematically embed “foresight throughout an entire governance architecture, including policy analysis, engagement, and decision-making” ([47], 3).

Most related work has started by outlining institutional and organisational aspects. At the European Union [11], the first Strategic Foresight Report establishes strategic foresight into the regular agenda, perceiving anticipatory governance with a new sense of urgency. The second Strategic Foresight Report [12] highlights open strategic autonomy. In OECD [48], foresight is recommended as a tool for successful policy-making in the face of high uncertainty. In the UN, strategic foresight is integrated by the UN Secretary General, as one of five key areas, in the report Our Common Agenda [63], presented to the UN Member States. At the national level, Finland’s Committee for the Future in Parliament, is currently a forerunner as the first permanent parliamentary standing committee dedicated to future matters in Europe. Koskimaa and Raunio [31] observe that despite an acute need for actualising and advancing anticipatory governance, establishing such units through legislature takes time. There is room to improve the role of citizens in AG efforts [25].

Given how we think, conceptualise and approach futures can translate into attempts to govern the future, AG should consider in detail of *how* precisely foresight is made use of. Muiderman et al. [45] explore the relationship of different conceptions of the future and their implications for the present. We specifically call for AG initiatives to embrace hybridisation with a view to emerging issues, weak signals, and their ambiguous nature. Without conscious efforts to integrate hybridisation into AG, it would seem these features are absent from governance efforts aimed and designed at addressing many types of future-related issues, including crises, and their ramifications. This makes unveiling the mysterious nature of hybridisation particularly interesting with a view to the Millennium Project 15 Global Challenges [19] and the United Nations Sustainable Development Goals (UNSDGs) [62].

Recent rapid changes and a number of events coined as crises, lacking adequate governance measures, have raised suggestions to integrate crisis awareness as part of futures literacy [30]. We suggest that crises reflect a VUCA world, as they have become more rapid and combined. With a view to the Climate Emergency [7, 28], Vervoort and Gupta [65] link AG and foresight with climate change, hoping for anticipatory climate governance, to see “the evolution of steering mechanisms in the present to adapt to and/or shape uncertain climate futures”. New crises unlike any we have witnessed before are also unfolding. The evolution of Artificial Intelligence (AI) into its elaborated forms Artificial General Intelligence (AGI) and Artificial Super Intelligence (ASI) points to an ontological crisis of the human-technology relationship. There will be crises we will fail to anticipate and struggle to imagine (“unknown unknowns”). The crisis of the imagination is connected to the ‘crises of the future’, where the ways to deal with uncertainty have not yet been imagined [42]. We may even be facing mega-challenges, mega-risks, and mega-crises. Especially in post-normal times [56], blinded by mega-challenges and mega-crises, a new modus operandi that recognises hybridisation, crises, their ramifications and wide-reaching implications, is called for. Bearing in mind the chance of existential risks [44], we only need one of them to be realised to find ourselves as a humanity in a mega-crisis. Are we already in a ‘crisis society’?

## Data and methodology

We adopted *hybridisation* and *open futures* as lenses for testing and ethically reflecting on *futures images* [52] conveyed by *hybrid urban spaces* as pioneering architecture**.** The aim of this heuristic was to imagine the possible consequences of various developments in the urban space. We identified contemporary and famous examples of hybrid urban spaces and imagined their future multiplication and divulgation. We also discuss crisis awareness and resilience with the context of hybrid urban spaces. Building on our literature review, we applied a hybrid methodology of pioneer analysis, posi- and nega- trend analysis and open/closed futures. Next, we present the methods and data used by drawing on three urban studies projects with foresight.

We elaborate the findings from two previous research projects on liveable urban futures. The foresight part of the ELOISA project, an abbreviation in Finnish from “Resilient Suburb”, of the Tila programme at Tekes (the Finnish Funding Agency for Technology and Innovation, now Business Finland) in 2011-2013, carried out as three Futures Cliniques, discovered three emerging issues: (1) environment and living of meaningful experiences; (2) local democracy and grass-roots approaches, and (3) hybrid spaces combining different functions. The ENCORE project (Finnish abbreviation from “Economically viable city centre and urbanising downtown”), funded by Turku Urban Studies Programme (2015–2016), elaborated how these issues act as drivers for urban liveability and economic viability. One finding proposed an anticipatory lens and a tentative conceptual model for renewing urban governance. A new layer and impetus to governance arises from research in the RESCUE project (“Real estate in sustainable urban crises management in urban environments”), examining risks and crisis in the built environment.

The pandemic as a crisis context and climate change, as a looming mega-crisis, give a new meaning to ‘a liveable city’. A city is not liveable unless it is crisis-proof i.e. ‘liveable’ even in times of crises. We wish to advance this approach by highlighting the hybridisation lens, crisis awareness and governance considerations. A focus on hybrid spaces within the urban socio-cultural texture makes for a meaningful milieu. At a time when new perspectives are searched for making cities sustainable and resilient through anticipation and awareness of crises, we explore whether hybridisation can support these objectives. We were interested in how hybrid urban spaces might articulate, complicate or express interpreting, overcoming or evading crises. Already, diverse typologies of crises and how they affect the urban environment have been identified (Tähtinen L, Toivonen S, Rashidfarokhi A: Built Environment Amidst Future Crises: Thematic Categorization of Crisis Impacts and Requirements for Building Resilience, submitted). Adaptable architecture is needed, but hybrid urban spaces have not been fully explored [50, 51].

The nature of hybrid urban spaces relating to their potential, promises as well as pitfalls and perils was revealed, drawing on the method of posi- and nega-trends [21]. The Open Futures framework [5, 27, 61] helps encountering a new innovative proposal or situation. It enables asking whether it opens up or closes futures, that is, what futures are being opened for whom, and what futures are closed for others. Further questions may entail ‘what unconceived futures are being closed’. In other words, do current processes and progressions leave the future open for more preferable solutions or better futures not yet invented or conceived? As illustrative cases [66] of forerunner architecture, three hybrid spaces as pioneers of the built environment were chosen, as an application of pioneer analysis [22], to envisage their diverse futures, what is opening (or closing) in them, and to leave room for futures that have not yet been anticipated.

## Hybrid urban spaces and their potential for future insights

Hybrid spaces combine different functions, aesthetics, and spheres of life, by fusing old and new, public and private, housing, leisure and work or digital, virtual and physical [24]. We discuss hybrid spaces in urban socio-cultural design and texture. We describe the functions, services and actions within such spaces to their users as well as the manifestations and meanings that these spaces are conveying. First, attention is given to three hybrid spaces in light of our four categories (characteristics, fields, functions, roles). Second, we reflect the role of hybrid spaces in the times of crisis. We assume them to buffer against crises *or* to contribute to their complicatedness. These mysterious faces of hybrid spaces are analysed to open up possible transformations, and to de-mystify them.

An architectural perspective is a common perspective to hybrid spaces, alluding to their multi-functionality. In architecture, time and hybridity relate to how buildings over time are used and adapted. For established, specific-use buildings, there is a constant contamination from the functions of the different spaces and facilities. Seasonal changes and short-term cyclical use “can lead to renewed architectural imagination” ([51], 272). Hybrid spaces in the built environment can be explored for ecological, spatial and mixed use adaptability. Pelsmakers et al. [51] state that their hybrid features have been under-explored despite their temporal nature. If complex temporal aspects are overlooked, the design for long-term ecological sustainability can ignore short-term needs. The inhabitants are even encouraged “to take over, to change the architecture, at different levels, and over time periods …” (ibid).

In turn, urban planning emphasises urban renewal, urban landscape development, and enhanced contributions to the urban economy [32]. Green spaces within the built environment create cohesive landscaping, place-building and identity. *Place attachment theory* [17, 57] explores how people attach value and can even make lifelong decisions through personal experience around certain places. As home, work and community are increasingly merging, the value of places as experience, constructed heritage, or life story, should be recognised. The attachment to places correlates to the meanings given to buildings and their environment, based on experiences and impressions of use. We emphasise how buildings can also be interpreted as metaphors that represent complex forms [23], as signs of future change, ideas for new directions, and different futures. However, it is not clear whether hybrid spaces signal future developments, and if so, how.

The performative nature of places, and hybrid spaces, can be interpreted through their symbolic power. Preziosi [53] describes aesthetics and power in ancient Greece, and how the *Periclean Acropolis* exerts power upon the onlooker who upon walking up the pathway would be exposed to a series of stages. A city that mixes power, aesthetics, religion and space reveals the many faces of the goddess; “she becomes pantheon in her own right, a metaphor of the city, and the acropolis multiplies Athena across a web of synecdoches.” (Ibid.). Philosopher Jeremy Bentham (1748–1832) designed the Panopticon circular prison that binds the individual or prisoner with panoptic gaze. Through Michel Foucault’s [14] work, panopticon has become a quintessential illustration of how power operates within society. Preziosi posits that spatial power is complex and contradictory, reminding us of how history, governance, values and power operate through spaces, as the interplay of pasts, the present and futures.

Liminal spaces as spaces in-between or even the ‘Non-place’ [2] are of particular interest from a futures studies perspective. Unlike places grounded in history and social culture, synthetic modern spaces like supermarkets or airports change our very sense of perception, as socially sterile, highly functional and information intense, as qualities that clarify the sense of disconnect. Similarly, recently constructed digital worlds further detach from reality owing to their own logics, rules and behaviours, becoming a Metaverse. Although difficult to define, even these emergent non-places may over time manage to find further grounding in new contexts. These above examples illustrate some of the diverse ways of how hybrid spaces are encountered in the urban texture, which we have taken into consideration in studying them.

As our interpretive lens, the Open Futures approach guides us in evaluating the intentions and implications of hybrid spaces for futures [61]. We might, for example to elaborate this analytical strategy, consider a hypothetical design and construction of a shopping centre on a plot of unused parkland. From a planning perspective, the building is designed with specific energy needs, for specific stores with assumptions of financial viability and return-on-investment for a thirty-year timeframe. From our perspective, such assumptions do not account for the change dynamics, the value of the habitat or diverse future alternatives. Therefore, implementing a plan is as much about the building per se, as it is about determining where people will be physically located and earn their livelihood. Most importantly, it is, a lock-in to a specific future, which extends far beyond the next 30 years, and closes the future for this park environment. Questions are raised at multiple levels: how and for whom are futures opened or closed? Furthermore, what unrealised, even unknown futures are narrowed, closed—or opened? As a foresight approach, we find value in the consideration of hybrid spaces for exploring governance challenges, coping with crises, to offer insights that encourage open futures that are also desirable.

### Three faces of hybrid spaces

**Fig. 1:**
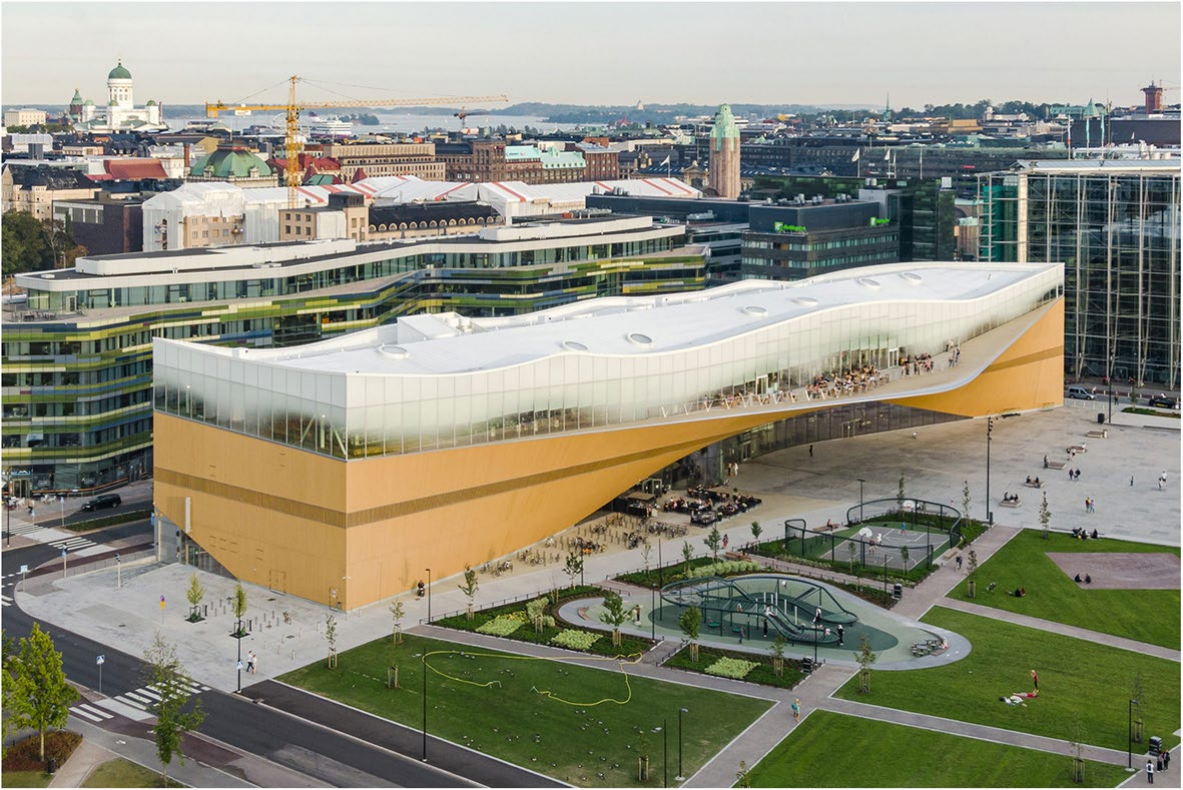
Oodi Library, Helsinki

The many faces of crises require visiting the many faces of hybridisation. We present three pioneering architecture cases of hybrid spaces, as *three different faces* that illustrate and open up hybridisation and crisis resilience in the urban context. The purpose of presenting these three cases is to analyse examples of hybrid spaces concerning what kind of futures images they point to and to which futures they are opening or closing. Thus, we also learn about their resilience to crises (Fig. 1).

*The Oodi Library* is a hybrid of a library, public living room and maker space, as a catalyst for social wellbeing and equality. It is a highly versatile building in ample space, proposed as a ‘living room’ for the population of the city of Helsinki and its guests. Primarily, it is an oversized city library opened in 2018. As a library, it stretches its function to offer maker spaces, events and workshops, plus a small cinema. Located in the heart of the capital, opposite the Finnish Parliament building, alongside a music concert hall and a sprawling public square, it is identifiable as a symbol for the welfare state. It encompasses multiple stories and has different functions for events in its open space. The second level has multiple meeting and workshop spaces, and the top level offers a landscape view, an extensive book collection, live trees, a café, and robots transporting the books. It promotes EU activities, houses a citizen advice service, enables socialising, leisure, learning and exploration. Above all, it is open for everyone. The deck view from the upper level of the sweeping wooden façade looks over a public square where skateboarders are encouraged to play (see [1]) (Table 2).

**Table 2:** Anticipatory analysis of the Oodi Library

**How is it a hybrid space**? • A fusion of learning and entertainment, technology and culture. • A multifunctional space, potentially versatile enough to adapt to any future need, while at the same time functioning as a traditional library. • Reimagines what the library can be, as a social catalyst and support system for the wellbeing state.	**What futures are opening or closing?** • Opening futures for equality. • Closing futures for exclusive privatised or restricted spaces.
**What insights about the future does it present, particularly concerning crisis?** • As cost of living in Finland is quite high, the need for comfortable safe and thriving living spaces, are extended beyond private homes. The public spaces become extensions of living spaces, that can be considered a *posi-trend*, also emphasising peer to peer approaches. • Tackles social marginalisation, loneliness and isolation, centralised to create a citizen space rather than a commercial urban space. Individual learning is encouraged, embracing maker-movement style problem fixing. For example, you can borrow a sewing machine or use a 3D printer. • A *nega-trend* may arise from this extreme openness with risks of criminal or antisocial behaviour. In times of crises, the facilities can quickly be modified to accommodate various functions and for all walks of life.	**What anticipatory governance does it provoke?** • The welfare state, education and the urban experience is democratised, a mechanism for equality for knowledge and culture.
**What image of the future of a city does it imply?** • *A city of communicative wellbeing*: a city that is equally open and accessible to all users. • A city where people and places interact and communicate, in concrete and symbolic terms.	

**Fig. 2:**
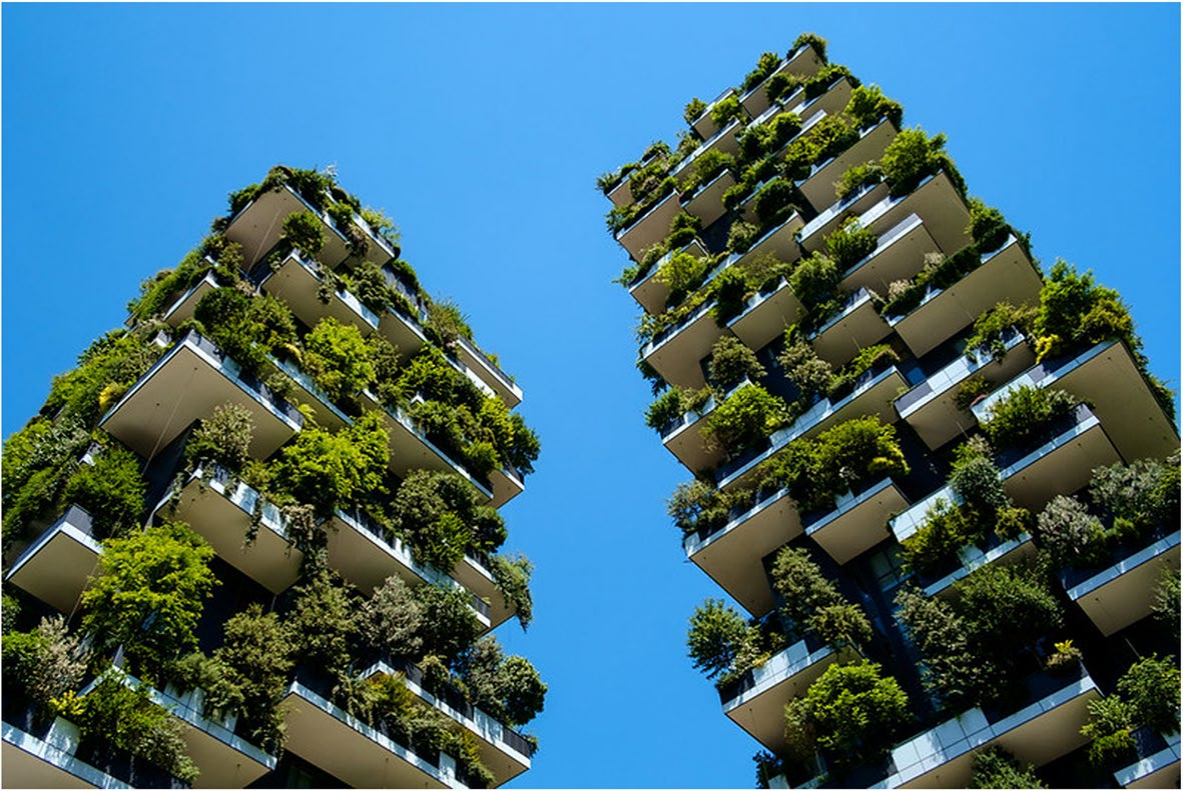
Bosco Verticale, Milan

*Bosco Verticale in Milan, Italy* has emerged as a modern classic of green ecological construction (Fig. 2). As a Vertical Forest, it is literally a hybrid of forestry elements in a few high-rise apartment buildings. The project also affects the city as a whole, as a new quarter and a green space circling the city. The eco-conceptualisation of Milan, i.e. making it into a BioMilano, aims to build a green city of the future, where newest environmental technology and emerging trend-based solutions, such as vertical farming, are utilised. Bosco Verticale is one of the projects in the programme meant to clean up Milan’s reputation as a polluted city. The metaphor is a forest—not just a park or garden, so that people can lead modern, urban lives in the middle of a refreshing forest (Table 3). Entire trees, not just green plants, grow on the balconies of the tall residential buildings, inviting pedestrians and cyclists. [23]. The architect firm proposed a similar type of city-wide greening project for Mexico City, Mexico and a coronavirus-resilient neighbourhood in Tirana, Albania (see [16, 55]).

**Table 3:** Anticipatory analysis of Bosco Verticale

**How is it a hybrid space**? • A hybrid of technology and nature. Integrating nature as a component within the built building concept, with the added benefit of forest and vertical farming. • Combining state of the art green technologies into traditional construction within city centres.	**What futures are opening or closing?** • Urban green healthy futures are opening, as potential to apply radically green technologies and practices to redefine architecture and living. • Potentially closing concrete based buildings—buildings that do not have a greening function might face limited futures.
**What insights about the future does it present, particularly concerning crisis?** • Tackling urban pollution and climate change, the project is also a pioneer case for how cities could be more ecological. • A posi-trend could result in nature being easily and holistically integrated, ultimately approaching biomimicry. • A nega-trend could be a superficial application of natural elements, contributing to greenwashing. • In times of crises such as pandemic or high air pollution, residents locked in their apartments still have the sense of greenery surrounding them.	**What anticipatory governance does it provoke?** • Construction, usually with negative connotations for climate change footprint is here reversed where it becomes profitable to build healthy buildings.
**What image of the future of a city does it imply?** • *City of fluid urban forest*: a city inviting the whole forest concept not just green areas to become an integral part of the city texture. • Positively contagious viral large-scale green urban designs.	

**Fig. 3:**
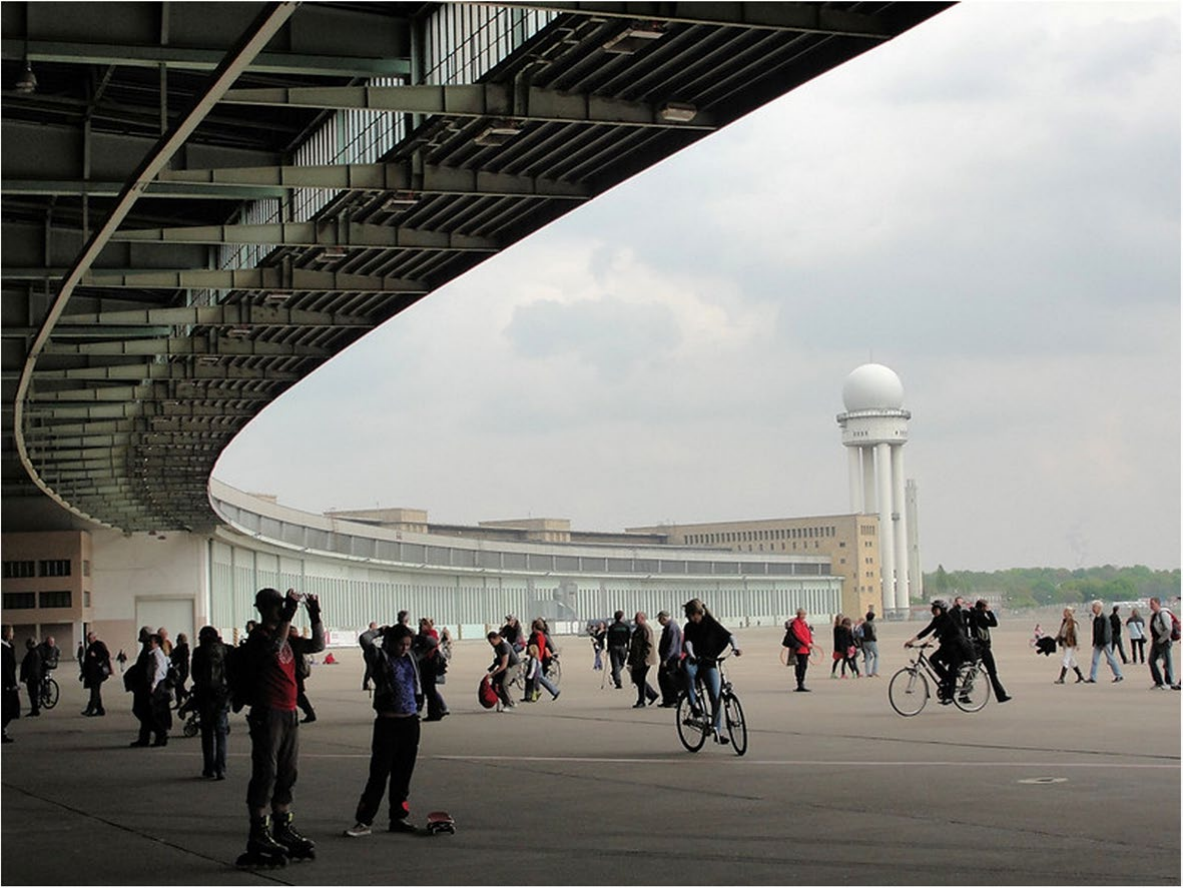
Tempelhof Airport, Berlin

*Tempelhof Airport* in Berlin represents a space with a strong history (Fig. 3). The Airport, initially a strategic development of the Nazi regime, has been a significant site before, during and after the Second World War. The disused Berlin airport was set for developers to take over, but after local Tempelhof citizens voted to stop building development, recent years have molded it into a cultural hot spot. It has become utilised for cultural and temporary events, like concerts, festivals, and restaurants. An acute need arose during the refugee crisis in 2015 where Germany had a sudden influx of migrants. This historical site and its spaces offered temporary housing for migrants as a camp. Its large buildings were quickly adapted to make living cubicles. A solution-oriented approach during a crisis brought controversy due to the strict restrictions of such historical buildings use, as the migrants attempted to customise and improve their basic living spaces during their stay. The adaptation for migrants from a WWII Nazi era symbolic space, prone to incubate political confrontation, shows the politics of spaces, whereas economic interests may also prevail over the flexible adaptation of space for cultural events, or for temporary shelter. Both hybrid features and complex power relations point to *entangled* futures, where urban development needs clash with cultural freedom and protection of vulnerable groups [49] (Table 4).

**Table 4:** Anticipatory analysis of the Tempelhof Airport

**How is it a Hybrid space**? • Highly adaptable, while grounded in history, versatile for emerging situations. • A temporal hybrid where intertwined past, present and future connotations are looming. • A hybrid of public and private (even refugee camp is a private space), and of housing and mobility.	**What futures are opening or closing?** • Open futures appear as supporting human migration from desperate parts of the world, becoming new German and Europeans, with local adaptable culture. • The closed futures for the airfield as a cultural hub derive from disruptions, the closing of cultural event path can lead to short-sighted property development taking over, a political window of change is shrinking.
**What insights about the future does it present, particularly concerning crisis?** • Migration crises are not necessarily creeping crisis, but may occur suddenly. Coexistence of acute temporary human need within a space with culture can thrive. • A *posi*-trend is the agile adaptation of governance and use of city space, transferring from one use to a totally different one. • A *nega*-trend can be discerned in the forced unsatisfactory compromises. In times of crises, technical flexibility to function as a buffer is diminished by possible political disputes.	**What anticipatory governance does it provoke?** • Local and national positive agile reaction is possible propelled by urgency of crises, expressing global responsibility. • Grassroots movement enabled future positive platforms for resilience while maintaining heritage futures.
**What image of the future of a city does it imply?** • *A city of stellar solidarity*: City authorities are hand in glove with grassroots movements and their cultural activities and initiatives. • While in case of bigger emergency like refugee influx, agile solidarity has to be put in place.	

## Improving anticipatory work by interpreting hybridisation

This section discusses ways to integrate interpretations of hybridity in futures studies and foresight. We then discuss ways in which learning from hybridisation could enhance our preparation to futures, as a lever for the construction of futures, in identifying crises, alleviating them, and in opening and closing futures, drawing on our case study of urban planning, and related governance efforts.

We propose (at least) five different uses of introducing hybridisation in futures studies. First, when foresight helps make visible the early signs of changes, interpreting hybridisation could be integrated into these efforts, assisting us to come to terms of the prevalence of different types of novelties in society, and to make sense of them. We claim that recognising hybridisation helps provide a picture of our complex world, instead of idealised or outdated decision-making frameworks and models. Second, learning from the different kinds of hybrids of a changing world may point to other types of emerging issues, which have not yet been properly conceptualised. In practice, on both counts, interpretations of hybridity could become an aspect of deeper attention when identifying and interpreting weak signals. Third, such attempts that learn from the diverse facets of emerging issues through hybridisation could make use of the proposed categories. Hybrid features can be studied in multiple fields, in different actors, entities or issues who are placed at multiple levels, scales and places. Fourth, an explicit concern with hybridisation could be used as a way to explore and embrace challenges and uncertainties, instead of shying away from them, to open up related possibilities. Fifth, examining hybridisation may assume a view to potentially growing impacts of emerging issues. They may offer a view to looming issues in the horizon, if entirely unaccounted for (and the possibility of crises ahead). Therefore, we claim as a specific segway that hybridisation can offer partial assistance in enhancing our crisis literacy.

With respect to interpreting hybridisation, as fusions that may help anticipating urban futures, our three examples were tested as regards the insights of future cities they may imply. Using rigorous imagination [38] we evaluated all three as possible future prospects in urban development. Their probability does not differ much. We queried whether they reflect preferred images of the future. The three pioneering architecture examples point to potentially positive and negative developments. The heuristics of hybridisation and open futures help testing and ethically reflecting on scenarios, futures images and ascribed imaginaries. The futures images that these spaces evoke are about ethical evaluation—meaning here for example asking, if they are closing futures for some group of citizens. In practice, actors could imagine the possible consequences if some types of hybrid spaces will increase in the built environment, detecting what is opening (or closing) in them.

Cities are central places for all types of adaptation [35]. For urban planners, we offer a new meaning and a deeper view that can help make sense of hybrids, as a way of interpreting to what futures these ‘hybrid spaces’ are pointing to. A useful test is on the potential of these hybrid spaces to deliver wellbeing and resilience in the urban texture. An urban context as an entry point to hybridisation is about the actualisation of governance that assumes an anticipatory stance to empirically learn from hybrid urban spaces. When the proposed approach is applied or adapted in urban planning and municipal level, it should start from imagining the alternative consequences of various developments. As part of horizon scanning, it would be possible to identify and analyse existing hybrid spaces, their hybridity, pros and cons, and openness or closedness for futures. Related questions, aiming at a futures-conscious approach to help shape the urban agenda, could entail whether hybrid spaces in the urban context are serving desirable values, are opening or closing futures, or seem crisis-resilient. Such efforts would begin to inform practical questions on which actors should be involved, how, and with what types of interventions. Again, as reminded by our cases, including citizens at the heart of these processes is important to succeed in such efforts.

By showing a way to meaningfully interpret hybridisation, as the signs of fusions of emerging issues, is about addressing a core challenge, namely “how to act in the present”. Despite recent calls to introduce a futures approach to governance, as anticipatory governance, there have been limited efforts that holistically incorporate the facets of hybridisation in anticipatory frameworks. In our view, information from the pioneers and weak signals embodying the fusions that characterise futures must be translated into actionable and strategic responses through deliberation. We call for futures dialogues that engage various stakeholders: city authorities, developers, investors, business angels, citizens, artists, and media. In bringing them together, such deliberative foresight may concern questions, such as (1) what kinds of hybrid spaces and places are needed, desired and by whose design, (2) how they can be implemented creatively and innovatively to guarantee resilience and protection against crises, and (3) how the city will become more liveable, resilient and attractive because of such hybrid spaces? We claim that engaging with hybridisation can improve awareness of the combinations of distraught sets of issues, and how they might combine. If we are right, we may build capacities to improve governance, including understanding on the causes of crises, their impacts, and resulting futures.

## Conclusions

In this paper, we have set a scene for scholarly work in futures studies to take note and to address hybridisation. We claim that when foresight engages with hybridisation, as a previously overlooked topic, it can generate meaningful views that point to futures. As viewed through hybridisation, a concern with present developments helps considering how, for example, our contemporary environments and our relationship to those environments, are constantly shaped. In our view, being attentive to hybridisation is important because of constantly and increasingly rapidly changing contexts of the present day VUCA world, full of volatility, uncertainty, complexity, and ambiguity. In practice, we point to the necessity to explore the novel expressions of hybridisation and integrate them into foresight activities.

When we opened up the phenomenon of hybridisation, we focused on its role in urbanising societies. Toivonen et al. [60] already discuss the necessity of empowering city developers through futures literacy. Cities, as venues of vast imagination, were explored through special attention directed at hybrid urban spaces, with a tentative typology, and reflections of these spaces. Hybrid spaces have mysterious faces—their manifestations may be resilient and protect in times of crises, opening up positive images of the future. Sometimes, their implications are negative and difficult to govern. This ambiguity has to be de-mystified to benefit from the potential of building resilience. Therefore, further study is needed on the transformative potential of hybrid urban spaces. When applying the futures lens on hybrid spaces, attention can be drawn to whether and how they implicate open or closed futures—embedding opportunities or restrictions. As interesting topics in further studies on futures of hybrid urban spaces, we highlight their temporal aspects, co-productive stance, as well as their dual attraction to users and urban developers.

Building on this paper, futures studies and foresight practitioners can continue to explore hybridisation, and its diverse manifestations. To sum up, the core contribution of our article to the field of futures studies is three-fold: (1) introducing the understudied topic of hybridisation within the framework of the VUCA world; (2) adding hybridisation as an element to futures literacy, with the aid of a typology created for different categories of hybridity; and (3) proposing hybridisation to be embedded into anticipatory governance, as a novel, enhanced framework to be used, particularly in urban planning. As a new element in efforts aimed at anticipatory governance, thinking with hybridisation is about versatile preparedness, crisis awareness, and innovative solutions. Further work that supports liveability and sustainability is about paying attention to how futures are opened and closed, and offering a way forward that obtains futures resilience.

## Data Availability

None

## Footnotes

1 See also: https://www.houstonforesight.org/futurists-vs-strategists-the-fusion-of-both-roles-to-survive-in-the-vuca-world/
